# Comparison of the Modulatory Effect on Intestinal Microbiota between Raw and Bran-Fried Atractylodis Rhizoma in the Rat Model of Spleen-Deficiency Syndrome

**DOI:** 10.3390/ijerph16173183

**Published:** 2019-08-31

**Authors:** Shanpeng Ma, Yujun Jiang, Beixue Zhang, Jian Pang, Xiaoying Xu, Jianzhi Sun, Xin Lv, Qian Cai

**Affiliations:** Department of Medicine, Liaoning University of Traditional Chinese Medicine, Dalian 116600, China

**Keywords:** Atractylodis Rhizoma, bran-fried, spleen-deficiency syndrome, intestinal microbiota, 16S rDNA, mechanism

## Abstract

Atractylodis Rhizoma (AR), a kind of well-known traditional Chinese medicine (TCM), has a long history of being used to treat spleen-deficiency syndrome (SDS). Stir frying with bran is a common method of processing AR, as recorded in the Chinese Pharmacopoeia, and is thought to enhance the therapeutic effect in TCM. Our previous studies have confirmed that bran-fried AR is superior to raw AR in terms of the improvement of gastrointestinal tract function. However, the biological mechanism of action is not yet clear. Here, we report the difference between raw and bran-fried AR in terms of the modulatory effect of intestinal microbiota. We found that the composition of intestinal microbiota of SDS rats changed significantly compared with healthy rats and tended to recover to normal levels after treatment with raw and bran-fried AR. Nine bacteria closely related to SDS were identified at the genus level. Among them, the modulatory effect between the raw and bran-fried AR was different. The improved modulation on *Bacteroides*, *Escherichia-Shigella*, *Phascolarctobacterium*, *Incertae-Sedis (Defluviitaleaceae Family)* and *Incertae-Sedis (Erysipelotrichaceae Family)* could be the mechanism by which bran-fried AR enhanced the therapeutic effect. Correlation analysis revealed that the modulation on intestinal microbiota was closely related to the secretion and expression of cytokines and gastrointestinal hormones. These findings can help us to understand the role and significance of bran-fried AR against SDS.

## 1. Introduction

Spleen-deficiency syndrome (SDS) is a common clinical syndrome of traditional Chinese medicine (TCM). According to the TCM theory, the spleen governs the transportation and transformation in the organism, which is the foundation of acquired constitution and the biochemical source of *Qi* and blood [1]. The main functions of the spleen include digestion of food and water, absorption and distribution of nutrients, and transfer of metabolic waste [2]. Splenic hypofunction can result in the insufficiency of digestive and nutrient-absorbing function, low-level energy metabolism, decrease of disease resistance, and decline of the function of various tissues, etc. The clinical symptoms of SDS include emesis, indigestion, diarrhea, and edema [3,4]. Many modern gastrointestinal diseases, such as gastric ulcers, irritable bowel syndrome and functional diarrhea, belong to the category of SDS in TCM [5,6].

Atractylodis Rhizoma (AR), called CangZhu in China, is the dried rhizome of *Atractylodes lancea* (Thunb.) DC. or *Atractylodes chinensis* (DC.) Koidz. It has the functions of drying dampness, invigorating the spleen, dispelling wind-cold infections and improving eyesight [7]. AR, with a long medicinal history in China, was first recorded in the Shennong Herbal Medicine Classic and listed in the top grade. There are only two kinds of AR recorded in the Chinese Pharmacopoeia: one is raw and the other is bran-fried. Use of the raw and processed herb for different purposes is a distinct theory in TCM. According to TCM theory, bran-fried AR is considered to alleviate the dryness effect [8] and strengthen the spleen and stomach [9] to a greater extent than raw AR, and thus is often chosen to treat SDS in clinics.

In our previous research, we compared the difference between raw and bran-fried AR in terms of chemical composition, pharmacological action, absorption, distribution, metabolism, and excretion [10,11,12]. Yu et al. [13] found that bran-fried AR had more satisfactory effects in the treatment of gastric ulcers than raw AR. Wang et al. [14] also revealed that bran-fried AR was superior to AR in regulating gastrointestinal hormones. Interestingly, despite its use as an effective medicine to improve gastrointestinal function, there is a distinct lack of systematic research on the relationship between raw and bran-fried AR with intestinal microbiotas, which play an important role in the immune response, maintaining body metabolism and nutrient metabolism, and serving as a physiological barrier [15,16]. Studies have increasingly shown that there is a close relationship between SDS and intestinal microbiota [17]. Impaired digestive and absorptive functions in a person suffering from SDS can destroy the balance of their organs, which leads to an imbalance of intestinal microbiota. An imbalance of intestinal microbiota can affect nutrient metabolism and further aggravate the symptoms of SDS. Many Chinese medicines used to treat SDS have been reported to modulate intestinal microbiota [18,19]. However, it is unknown whether raw and bran-fried AR can modulate intestinal microbiota.

We speculated that AR could improve the intestinal microbiota imbalance caused by SDS and that the modulatory effect of raw and bran-fried AR was different. To prove this conjecture, we duplicated the model of SDS by a compound factor method, which was composed of poor diet, excessive fatigue and severe diarrhea caused by bitter and cold Chinese medicine. 16S ribosomal DNA (rDNA) gene sequencing technology was used to study the composition of intestinal microbiota in SDS rats before and after treatment with raw and bran-fried AR. From this, the processing principle of bran-frying could be explained by comparing the difference in the modulatory effect on intestinal microbiota between raw and bran-fried AR.

## 2. Materials and Methods

### 2.1. Plant Materials

AR, collected from the Herb Planting Base of Luotian (Hubei, China), was identified as the dried rhizome of *Atractylodes lancea* (Thunb.) DC. by Professor Liang Xu from the School of Pharmacy, Liaoning University of TCM (Dalian, China). Bran-fried AR was made in our laboratory using the same batch of raw AR in accordance with the method outlined in the Chinese Pharmacopoeia (General Principles 0213, Part IV, 2015). Specifically, 30 g of bran was put into a heated pot, which was hot enough to make the bran smoke immediately. As soon as possible, 200 g of AR was added to the pot and stir-fried quickly until the surface was dark yellow. The bran-fried AR was removed and cooled down, while the bran was sifted out. Voucher specimens (Raw number: 201806110201R; Bran-fried number: 201806110201P.) were deposited in the Herbarium of Liaoning University of TCM (Dalian, China). Both the raw and bran-fried AR were crushed into a fine powder and sieved through a 160 mesh stainless steel sieve. Raw and bran-fried AR powder suspensions (375 mg·mL^−1^) were then prepared with normal saline for reserve. Finally, senna leaf (Batch number: Z13020471), purchased from the Outpatient Department of TCM of the Affiliated Hospital of Liaoning University of TCM (Shenyang, China), was boiled for 30 min, filtered by gauze and concentrated into a decoction (0.5 g crude drug per milliliter) for reserve.

### 2.2. Animals and Groups

Specific pathogen Free (SPF) male Sprague Dawley (SD) rats (6 weeks old, 200 ± 20 g) were purchased from Liaoning Changsheng Biotechnology Co., Ltd. (license number: SCXK(Liao)2010-0001, Liaoning, China) and housed at a certified animal experimental laboratory with a 12 h light/dark cycle, a constant temperature of 25 ± 1 °C, and 55–75% relative humidity. Animals were allowed free access to food and water. After acclimatization for one week, the rats were randomly assigned to four groups (*n* = 10 per group): control group (CG), model group (MG), raw AR group (RA) and bran-fried AR group (BFA).

For all of the groups except the control group, the rat model of SDS was established via a compound factor method, which involved an irregular diet, excessive fatigue and severe diarrhea caused by bitter and cold Chinese medicine. For days 1–14 of the experiment, the rats were intragastrically administered lard (10 mL·kg^−1^) on odd-numbered days. On even-numbered days over this period, the rats were forced to swim exhaustedly and were only fed cabbage (freely). For days 15–21 of the experiment, the rats were intragastrically administered the senna leaf decoction (10 mL·kg^−1^). This model spanned 3 weeks, during which the sawdust bedding was wetted. From the beginning of the 22nd day of the experiment, the RA and BFA groups were respectively intragastrically administered raw and bran-fried AR powder suspension (3750 mg·kg^−1^) for seven consecutive days, during which the control and model groups were gavaged with saline daily. All of the studies on animals were approved by the Animal Ethics Committee of the Affiliated Hospital of Liaoning University of TCM (Shenyang, China).

Body weight and fecal moisture percentage were recorded on the first day of the experiment and on the last day of each week, and additionally recorded daily during the treatment with raw and bran-fried AR. The fecal moisture percentage of each group was measured by the method outlined in Ref. [20]. Briefly, the fecal samples of each group were weighed, dried in the oven at 80 °C for 24 h, weighed again, then placed in the oven again for 1 hour and weighed a final time. In this work, a constant weight was defined as a change in fecal weight which did not exceed 3 mg. The calculation formula of fecal moisture percentage used was: (wet weight—dry weight)/wet weight·100%.

### 2.3. Biochemical Analysis

When the administration was over, all animals were anesthetized by intraperitoneal injection of chloral hydrate (30 mg·kg^−1^) after overnight fasting. Abdominal aorta blood samples were collected and centrifuged under the condition of 4000 r·min^−1^ for 10 min at 4 °C. Serum was separated and stored at −80 °C until analysis. Enzyme-linked immunosorbent assay (ELISA) was used for quantitative detection of serum tumor necrosis factor-α (TNF-α), interleukin 6 (IL-6), immunoglobulin G (IgG), Na^+^/K^+^-ATPase, motilin (MTL), gastrin (GAS), trypsin (TRY), amylase (AMS), and somatostatin (SS). Each operation was carried out in strict accordance with the kit’s instructions. All kits were purchased from Shanghai Kexing Trading Co., Ltd (Shanghai, China).

### 2.4. Fecal Sample Collection and 16S rDNA Sequencing

Eight rats were randomly selected from each group, and 0.2 g of fresh feces was collected into a sterile 2 mL centrifuge tube. Genomic DNA was extracted from the fecal samples using a TIANamp Stool DNA Kit purchased from TIANGEN Biotech Co., Ltd (Beijing, China). A UV spectrophotometer and agarose gel electrophoresis were used for concentration and purity testing. DNA sample quality was available when the total amount of DNA was greater than 500 ng. The V3 and V4 region of the 16S rDNA gene was amplified with specific barcoded primers. The primer sequences were as follows: 341F: 5′-CCTACGGGNGGCWGCAG-3′; 785R: 5′-GACTACHVGGGTATCTAATCC-3′ [21]. The PCR product was then collected and quantified using a QuantiFluorTM fluorometer (Promega Corporation, USA). The purified amplification products were mixed in equal amounts and then sequenced to construct a sequencing library. The samples were sequenced with HiSeq2500 PE250 (Illumina, Inc., San Diego, CA, USA).

### 2.5. 16S rDNA Bioinformatics Analysis and Statistics

Off-line paired-end sequences were joined by FLASH software, after which unmatched, mismatched and low abundance sequences were removed by Mothur software. UCHIME software was used to remove chimeric sequences to obtain clean sequences. The sequences were clustered into operational taxonomic units (OTUs) according to their sequence similarity by UCHIME software. The threshold value of similarity was set at 97%; that is, the sequences with similarity higher than 97% were clustered into an OTU. Sparse sequences were discarded in the process of OTU clustering. The OTU sequences were systematically classified by an RDP classifier referring to the SILVA database (http://www.arb-silva.de/). The classification was based on Bergey’s taxonomy and the default threshold value was set at 80%. In a single sample, the microbial abundance at each taxonomic level was calculated by the percentage of all sequencing items to the total sequencing items at that level. Alpha diversity indices, including the Chao index and Shannon index, were calculated by Mothur software. OTU sequences were aligned to the SILVA database by PyNAST software. The weighted UniFrac distance matrix between communities was generated by QIIME software. Principal Co-ordinates Analysis (PCoA) of the weighted UniFrac matrix was carried out by R software. LEfSe software was used to analyze the difference in relative species abundance between the control group and model group. Bacteria with linear discriminant analysis (LDA) scores greater than 3 and *p* < 0.05 were selected as the biomarkers of SDS, for which the modulatory effect of raw and bran-fried AR were compared.

### 2.6. Correlation Analysis

Spearman correlation analysis between serum biochemical indices and bacterial abundance indices closely related to SDS was carried out by SPSS 24.0 software. *p*-value < 0.05 was considered as a significant correlation. All correlation coefficients were displayed in the form of heatmap by HEMI software.

### 2.7. Statistical Multiple Comparison

Multiple comparison analysis was carried out using SPSS 24.0 software. Since each sample was an independent random sample, we assessed differences by ANOVA if it came from a normal distribution population with equal variance, and performed a correction for multiple comparison by the Bonferroni method. If statistical data did not conform to normal distribution or homogeneous variance, differences were assessed by the Kruskal–Wallis *H* test. *p*-value < 0.05 was considered as a statistically significant difference.

## 3. Results

### 3.1. Behavioral Comparison of Rats

**Figure 1:**
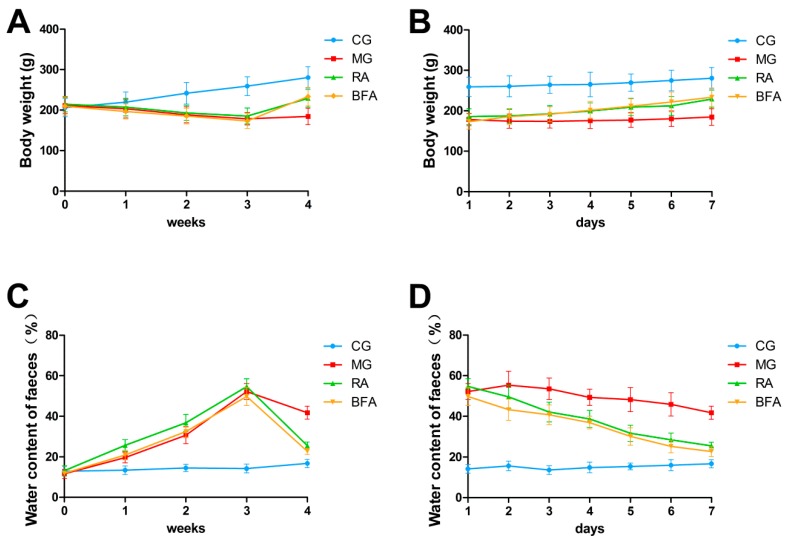
Comparison of body weight and fecal moisture percentage among diverse groups: (**A**) The curves of growth are based on the average body weight for each of the four groups during the whole experiment; (**B**) the curves show the changes of body weight for the four groups during treatment; (**C**) the curves of fecal moisture percentage are based on average values for the four groups during the whole experiment; and (**D**) the curves show the changes of fecal moisture percentage for the four groups during treatment. (*n* = 10 rats in each group).

The rats in the control group showed normal behaviors, including flexible movement and normal defecation, and had glossy fur and a growing body weight. In contrast, the rats of the SDS model group showed a series of abnormal behaviors, including lazy movement and squinting, and exhibited withered fur, an arched back, soft stool and a gradually decreasing body weight. After treatment with raw and bran-fried AR, the symptoms of the SDS rats were improved, and included an increase in body weight (Figure 1A,B) and the recovery of defecation (Figure 1C,D).

### 3.2. Effect of Raw and Bran-Fried AR on the Serum Levels of Biochemical Indices in SDS Rats

**Figure 2:**
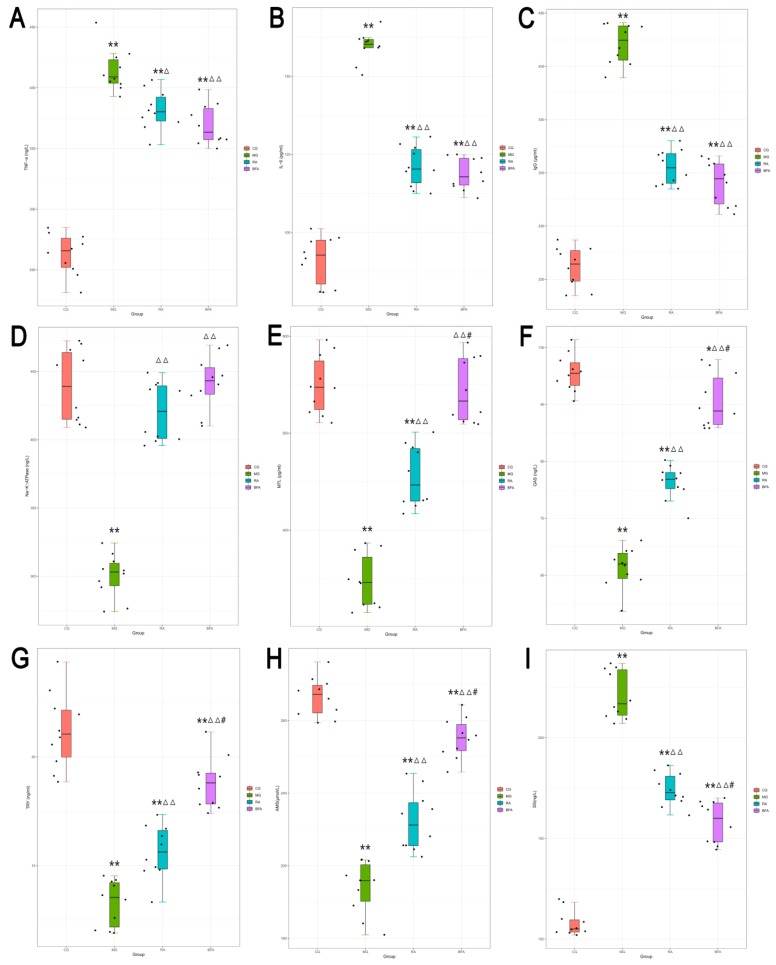
Comparison of the serum levels of biochemical indices among diverse groups: (**A**) Tumor necrosis factor-α (TNF-α) level; (**B**) interleukin 6 (IL-6) level; (**C**) immunoglobulin G (IgG) level; (**D**) Na^+^/K^+^-ATPase level; (**E**) motilin (MTL) level; (**F**) gastrin (GAS) level; (**G**) trypsin (TRY) level; (**H**) amylase (AMS) level; and (**I**) somatostatin (SS) level. Values are presented as box-plots, of which the black spots are the individual data points of each group (*n* = 10). Differences were assessed by ANOVA. ^*^ *p* < 0.05, ^**^ *p* < 0.01 when compared with the control group (CG); ^Δ^ *p* < 0.05, ^ΔΔ^ *p* < 0.01 when compared with the model group (MG); ^#^ *p* < 0.01 when compared with the raw Atractylodis Rhizoma group (RA).

Compared with the control group, rats in the SDS model group had significantly higher expression in the serum levels of TNF-α, IL-6, IgG, and SS (*p* < 0.01) (Figure 2A–C,I), and significantly lower expression in the serum levels of Na^+^/K^+^-ATPase, MTL, GAS, TRY, and AMS (*p* < 0.01) (Figure 2D–H). Compared with the model group, the raw and bran-fried AR groups exhibited significantly decreased serum levels of TNF-α (*p* < 0.05; *p* < 0.01), IL-6, IgG, and SS (*p* < 0.01) as well as increased serum levels of Na^+^/K^+^-ATPase, MTL, GAS, TRY, and AMS (*p* < 0.01) in the SDS rats. Among the AR treated groups, bran-fried AR was significantly superior to raw AR in regulating the serum levels of SS, MTL, GAS, TRY, and AMS (*p* < 0.01). Although bran-fried AR was also superior to raw AR in regulating the serum levels of TNF-α, IL-6, IgG, and Na^+^/K^+^-ATPase, there were no significant differences.

### 3.3. Comparison of Sequencing Depth and Alpha Diversity of Intestinal Microbiota from Fecal Samples in Rats

**Figure 3:**
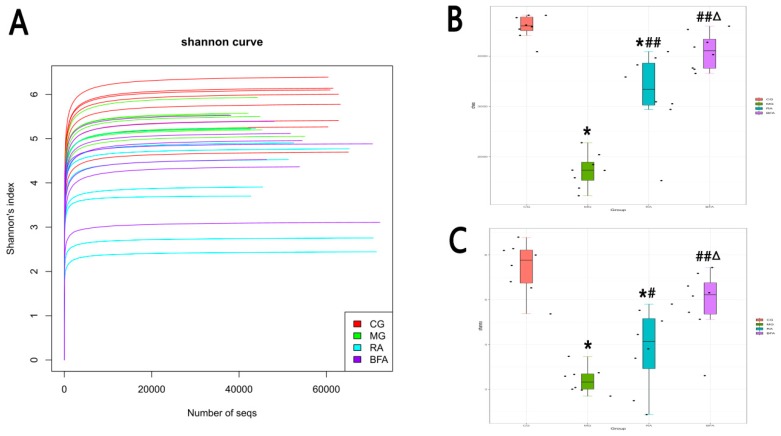
Comparison of alpha diversity of intestinal microbiota among diverse groups: (**A**) Shannon rarefaction curve of each group; (**B**) Chao diversity index of each group; **(C**) Shannon diversity index of each group. Diversity index values are presented as box-plots, of which the black spots are the individual data points of each group (*n* = 8). Differences were assessed by ANOVA. ^*^ *p* < 0.01 when compared with CG; ^#^ *p* < 0.05, *^#^*^#^ *p* < 0.01 when compared with MG; ^Δ^ *p* < 0.05 when compared with RA.

In the chart of the Shannon rarefaction curve, the curve of each sample tended to be smooth, suggesting the sequencing depth was sufficient to capture the biodiversity present in the samples (Figure 3A). Alpha diversity was reflected in the Chao index and Shannon index, indicating the richness and evenness of the observed species, respectively (Figure 3B,C). The control group had the highest level of bacterial diversity. The Chao and Shannon index values of the model group were significantly lower than that of the control group (*p* < 0.01). After the treatment with raw and bran-fried AR, the Chao and Shannon index values of these two groups were significantly higher than that of the model group. Furthermore, the Chao and Shannon index values of the bran-fried AR group were significantly higher than that of the raw AR group (*p* < 0.05).

### 3.4. Comparison of Beta Diversity and Abundance of Intestinal Microbiota at the Phylum Level from Fecal Samples in Rats

**Figure 4:**
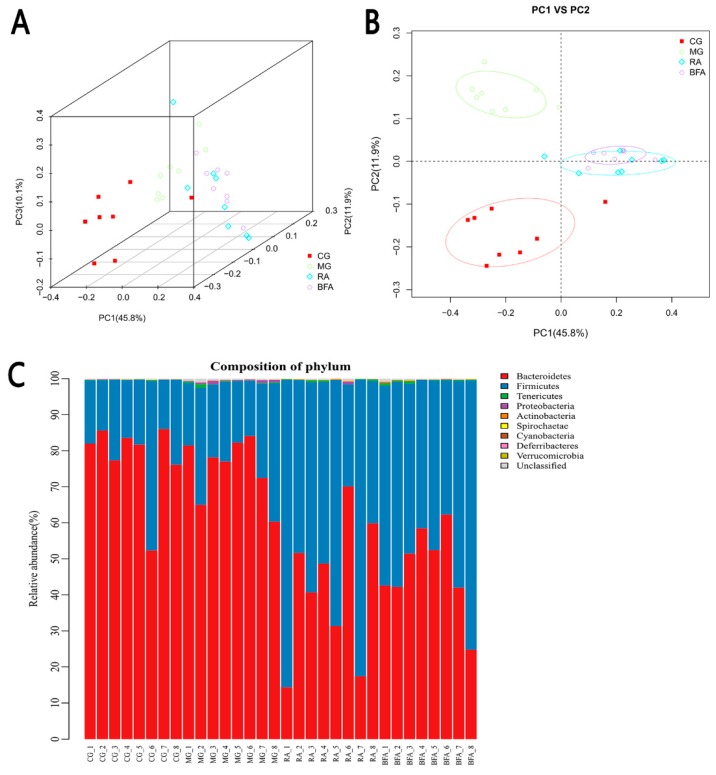
Comparison of the intestinal microbiota among diverse groups: (**A**) A 3D plot of the Principal Co-ordinates Analysis (PCoA) for the weighted UniFrac distance metric in intestinal microbial communities among diverse groups. (**B**) Principal Component (PC) 1 VS PC 2 of PCoA. (**C**) Histogram of bacterial flora distribution at the phylum level in each sample. Species that could not be annotated were classified into the ‘unclassified’ category. Differences were assessed by ANOVA.

The weighted UniFrac took into account phylogenetic distances between the OTUs, so that it captured differences not only at the level of individual phylotypes, but also at levels of higher taxonomic category. The weighted UniFrac PCoA (Figure 4A,B) revealed that the communities of the control and model group were separated completely, indicating that the intestinal microbiota composition changed significantly in the state of SDS. The communities of the raw and bran-fried AR groups lay between the communities of the control and model group, suggesting that the composition of intestinal microbiota in the SDS rats recovered to normal levels following treatment with the raw and bran-fried AR. Although the communities of the raw and bran-fried AR groups overlapped to some extent, there was a tendency to separate, indicating that the modulatory effect of raw and bran-fried AR may be different.

At the phylum level, we found that the majority of the intestinal microbiota consisted of species from *Bacteroidetes*, *Firmicutes*, *Tenericutes*, *Proteobacteria*, *Actinobacteria*, *Spirochaetae*, and *Cyanobacteria* (Figure 4C). There were, however, some differences among the four groups. For instance, compared with the control group, the abundance of *Proteobacteria* was significantly increased in the model group (0.057% abundance in CG, 0.452% abundance in MG, *p* < 0.01), while the abundance of *Spirochaetae* was significantly decreased (0.026% abundance in CG, 0.003% abundance in MG, *p* < 0.01). After administration of raw and bran-fried AR, the abundance of *Proteobacteria* and *Spirochaetae* tended to recover to the normal level. Specifically, the modulatory effect of bran-fried AR was found to be significantly superior to that of raw AR (*Proteobacteria*: 0.109% abundance in RA, 0.044% abundance in BFA, *p* < 0.05; *Spirochaetae*: 0.009% abundance in RA, 0.029% abundance in BFA, *p* < 0.01). Compared with the control group, the abundance of *Tenericutes* (0.152% abundance in CG, 0.313% abundance in MG, *p* < 0.05), *Actinobacteria* (0.023% abundance in CG, 0.049% abundance in MG, *p* < 0.05), and *Cyanobacteria* (0.011% abundance in CG, 0.002% abundance in MG, *p* < 0.01) changed significantly in the model group, although there were no favorable modulatory effects following administration of raw or bran-fried AR (*Tenericutes*: 0.291% abundance in RA, 0.374% abundance in BFA; *Actinobacteria*: 0.109% abundance in RA, 0.124% abundance in BFA; *Cyanobacteria*: 0.003% abundance in RA, 0.005% abundance in BFA). Additionally, as the dominant bacteria at the phylum level the abundance of *Bacteroidetes* and *Firmicutes* did not significantly change in the model group compared with the control group. However, the ratio of *Bacteroidetes* to *Firmicutes* significantly decreased after administration of raw and bran-fried AR (*p* < 0.05) (3.633 in CG, 3.37 in MG, 0.725 in RA, 0.902 in BFA).

### 3.5. Identification of the Bacteria Closely Related to SDS and Comparison of the Modulatory Effect on these Bacteria between Raw and Bran-Fried AR

**Figure 5:**
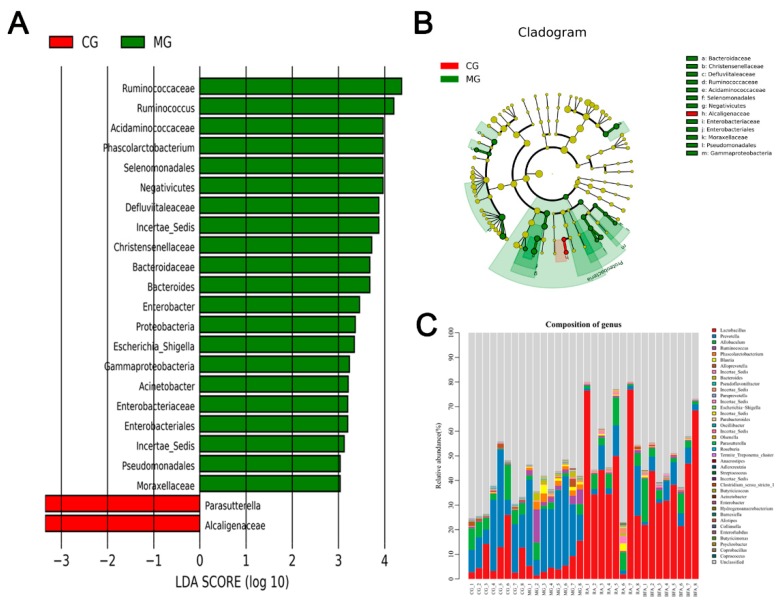
Identification of biomarkers of spleen-deficiency syndrome (SDS) and the abundance comparison of intestinal microbiota at the genus level among diverse groups: (**A**) Histogram of linear discriminant analysis effect size (LEfSe) analysis between the control group and model group. (**B**) Cladogram of LEfSe analysis. (**C**) Histogram of bacterial flora distribution at the genus level in each sample. Species that could not be annotated were classified into the ‘unclassified’ category.

Linear discriminant analysis (LDA) effect size (LEfSe) is a software that can perform linear discriminant analysis among different groups to find communities or species that exist at statistically significant differences. Typically, the horizontal axis is the LDA score value, while the vertical axis consists of taxa with significant differences among groups, ranked according to the size of their LDA score value. The longer the length of the bar graph, the more significant the difference is. Different colors of bar graph may be used to indicate that a specific taxon corresponds to a higher abundant sample group. On the basis of the result of LEfSe analysis between the control group and the model group (Figure 5A), nine bacteria at the genus level, including *Ruminococcus*, *Bacteroides*, *Parasutterella*, *Escherichia-Shigella*, *Phascolarctobacterium*, *Enterobacter*, *Acinetobacter*, *Incertae-Sedis (Defluviitaleaceae Family)*, and *Incertae-Sedis (Erysipelotrichaceae Family)*, were finally identified and considered to be closely associated with SDS. A cladogram was obtained from the LEfSe analysis of 16S sequences from each fecal sample. This cladogram, shown in Figure 5B, indicates the different microbial communities or species structures among the groups from phylum to genus (arranged from inner circle to outer circle). The size of the nodes correspond to the average relative abundance of the taxon. The yellow nodes represent the taxa that do not show significant inter-group differences, while the other colors (such as red and green) indicate that these taxa show significant inter-group differences and have higher abundance in the sample groups represented by the color. The letters identify the names of the taxa with significant differences between groups. A difference in microbial structure between the control group and model group was further evidenced by the results of this cladogram.

The abundance of all bacteria and biomarkers at the genus level among the four groups is shown in Figure 5C and Table 1. The abundance of the nine aforementioned bacteria closely related to SDS were found to all be regulated towards the normal level to varying degrees after administration of raw and bran-fried AR. Raw AR was significantly superior to bran-fried AR in the regulation of *Parasutterella* (*p* < 0.05). Conversely, bran-fried AR was significantly superior to raw AR in the regulation of *Bacteroides*, *Escherichia-Shigella*, *Phascolarctobacterium*, *Incertae-Sedis (Defluviitaleaceae Family)* and *Incertae-Sedis (Erysipelotrichaceae Family)* (*p* < 0.05). There was no significant difference between raw and bran-fried AR in the regulation of *Ruminococcus*, *Enterobacter* or *Acinetobacter*.

### 3.6. Correlation Analysis between Serum Biochemical Indices and Bacterial Abundance Indices Closely Related to SDS

**Figure 6:**
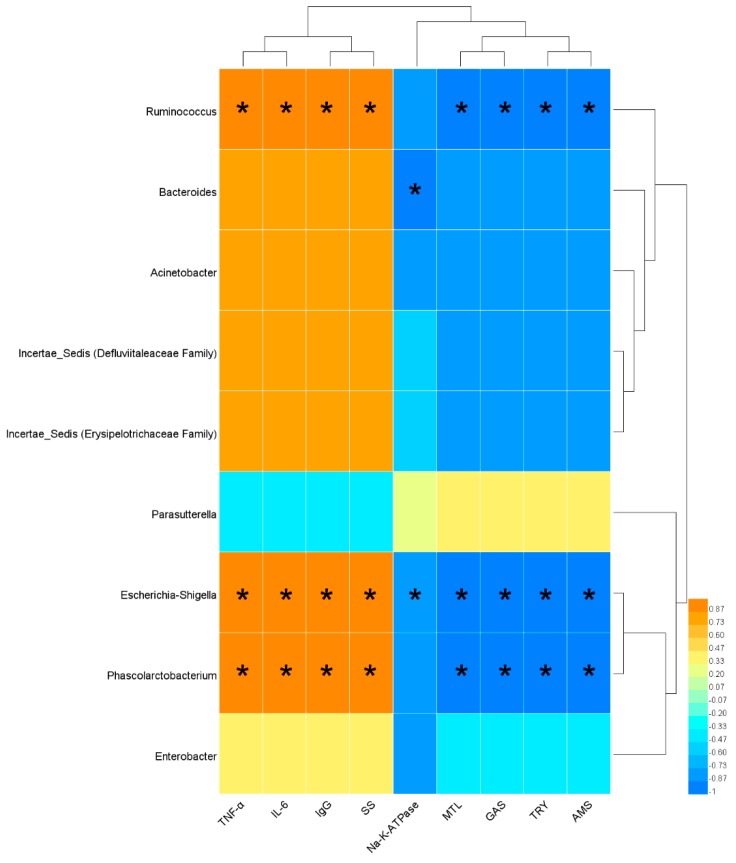
Heatmap of correlation coefficients between serum biochemical indices and bacterial abundance indices closely related to SDS. The x-axis indicates the serum biochemical indices and the y-axis indicates the bacteria closely related to SDS. Yellow represents positive correlation, while blue represents negative correlation. ‘*’ indicates a significant correlation (*p* < 0.01).

The results of the correlation analysis showed that *Ruminococcus*, *Escherichia-Shigella* and *Phascolarctobacterium* were significantly and positively correlated with TNF-α, IL-6, IgG and SS, and significantly and negatively correlated with MTL, GAS, TRY and AMS. In addition, *Bacteroides* and *Escherichia-Shigella* were significantly and negatively correlated with Na^+^/K^+^-ATPase (Figure 6).

## 4. Discussion

An animal model of a TCM syndrome is a kind of biological characterization model which uses some biological surface characteristics of an animal to simulate the characteristics of a human syndrome [22]. Correct replication of an animal model is an important guarantee to simulate the appearance and development of diseases. In this study, we duplicated the model of SDS by a compound factor method, which was composed of poor diet, excessive fatigue and severe diarrhea caused by the bitter and cold Chinese medicine senna leaf. The principle of this method is close to the etiological mechanism of TCM syndromes, and therefore it is generally adopted by researchers of SDS in China at present [23]. In this study, we found that the rats of the SDS model group showed a series of abnormal behaviors, including lazy movement and squinting, and exhibited an arched back, soft stool and a gradually decreasing body weight, which was in accordance with the diagnostic criteria of SDS in TCM clinics [24]. These characteristics were also consistent with the model of SDS duplicated by Pan et al. [25]. After administering raw and bran-fried AR, the symptoms of the SDS rats were improved, and included an increase in body weight and the recovery of defecation. This revealed that raw and bran-fried AR had certain effects on SDS.

TNF-α is a kind of cell factor produced by activated macrophages which can directly kill tumor cells, but has no obvious toxicity to normal cells. TNF-α is the fastest and earliest inflammatory mediator in the inflammatory reaction process [26], and can instigate inflammation by inducing the secretion of IL-6 [27]. IgG is the main component of serum immunoglobulin and the main antibody in vivo. It functions in anti-viral activity, neutralizing viruses, anti-bacterial activity and immune regulation [28]. In this study, we found that raw and bran-fried AR was capable of decreasing the serum level of TNF-α, IL-6 and IgG in SDS rats. The effect of bran-fried AR was superior to that of raw AR, but there were no significant differences.

Na^+^/K^+^-ATPase is a transmembrane carrier protein for ion transport in cell membranes. Its main function is to transport intracellular sodium ions to extracellular space and extracellular ions to intracellular space. This maintains the balance of electrochemical gradients of Na^+^/K^+^ inside and outside the cell membranes and maintains the balance of osmotic pressure inside and outside the cell. Furthermore, this provides conditions for active cell transport [29]. In this study, we found that raw and bran-fried AR could increase the serum level of Na^+^/K^+^-ATPase in SDS rats. The effect of bran-fried AR was superior to that of raw AR, but there were no significant differences.

GAS and MTL are two important gastrointestinal hormones, which are secreted by G cells and Mo cells, respectively. GAS acts on gastric parietal cells and promotes gastric acid secretion through the gastrin/cholecystokinin-B receptor [30]. MTL is distributed in the small intestine, and functions to promote and influence gastrointestinal motility and the transport of water and electrolytes in the gastrointestinal tract [31]. AMS and TRY are important digestive enzymes in the body with an essential role in nutrient absorption [32]. SS is a type of neurohormone, which can inhibit the release of GAS, MTL and TRY [33]. In this study, we found that raw and bran-fried AR could increase the serum levels of GAS, MTL, AMS and TRY, and decrease the serum level of SS. The effect of bran-fried AR was significantly superior to that of raw AR.

Through the detection of the above nine biochemical indices, we speculate that some of the mechanisms by which the raw and bran-fried AR acted against SDS included down-regulation of inflammatory factors, enhancement of immune function, and improvement of gastrointestinal digestion, absorption and water-liquid conversion function. One of the mechanisms which may have contributed to the enhanced therapeutic effect observed for bran-fried AR could be its significantly superior effect on gastrointestinal digestion and absorption function compared to raw AR.

In this study, through 16S rDNA sequencing, we demonstrated that treatment with raw and bran-fried AR could improve the disorder of intestinal microbiota caused by SDS. Compared with the control group, the diversity of intestinal microbiota in the model group was lower. Raw and bran-fried AR significantly improved the diversity of intestinal microbiota in the SDS rats. Through the analysis of intestinal microbiota composition, we found that, although the overall composition of intestinal microbiota in each group was similar, the proportions of microbiota within the composition were significantly different. At the phylum level, compared with the control group, the abundance of *Proteobacteria* in the intestinal tract of rats in the SDS model group increased significantly, while the abundance of *Spirochaetae* decreased significantly. *Proteobacteria* is a harmful bacterium which is considered one of the diagnostic markers of intestinal imbalance [34]. *Spirochaetae* is one of the predominant microbiota in the intestinal tract of organisms. The changes in these two microbiota indicated that the intestinal microbiota of SDS rats was in a state of imbalance. After the intervention of raw and bran-fried AR, the abundance of *Proteobacteria* and *Spirochaetae* tended to improve. Moreover, the modulatory effect of bran-fried AR was superior to that of raw AR. Thus, this may also be one of the mechanisms by which bran-fried AR enhanced the therapeutic effect. Additionally, we had an unexpected finding that, although the abundance of *Bacteroidetes* and *Firmicutes* in the model group did not change significantly compared with the control group, the ratio of *Bacteroidetes* to *Firmicutes* significantly decreased after administration of the raw and bran-fried AR. Both *Bacteroidetes* and *Firmicutes* are dominant bacteria in the intestinal tract. We speculate that the changes observed after administration may be related to the toxic and side effects of AR. Jiang et al. [35] also found proportional imbalance between *Bacteroidetes* and *Firmicutes* when studying the toxicity of Kansui. The principles of this change caused by AR remain to be further studied.

Based on the changes in composition of intestinal microbiota at the phylum level, we have a preliminarily understanding of the possible mechanism by which bran-fried AR enhanced the therapeutic effect, but it is not in-depth. As such, we further identified nine intestinal bacteria, such as *Ruminococcus*, *Bacteroides*, *Parasutterella*, *Escherichia-Shigella* and *Enterobacter*, at the genus level, which showed significant changes in their abundance in the state of SDS on the basis of a LEfSe analysis between the control and model groups. *Ruminococcus* and *Bacteroides* are bacteria that produce short-chain fatty acids (SCFAs)—such as butyrates—in the intestine. SCFAs play an important role in maintaining intestinal homeostasis. Butyrates are able to bind the G-protein coupled receptors GPR43, which are mainly expressed in the colonic epithelium, and thus can aid in maintaining normal intestinal permeability while suppressing mucosal inflammation [36]. *Parasutterella* occupies a specific intestinal niche and may be involved in the metabolism of bile acid and cholesterol [37]. *Escherichia-Shigella* and *Enterobacter* are common conditional pathogens. When the intestinal microbiota is balanced, they coexist harmoniously with beneficial bacteria. When the normal microecological balance is broken, they can cause endogenous infection [38]. The changes observed in these nine bacteria further illustrated the disorder of intestinal microbiota in the state of SDS. After intervention with raw and bran-fried AR, the abundance of these nine bacteria tended to return to normal. Thus, this may be one of the mechanisms by which raw and bran-fried AR exert their efficacy in the treatment of SDS. The correlation analysis revealed that the abundance of intestinal microbiota were closely related to the secretion and expression of cytokines and gastrointestinal hormones. They may interact to regulate immune function and gastrointestinal function. We found that bran-fried AR was significantly superior to raw AR in the regulation of *Bacteroides*, *Escherichia-Shigella*, *Phascolarctobacterium*, *Incertae-Sedis (Defluviitaleaceae Family)* and *Incertae-Sedis (Erysipelotrichaceae Family)*. This may therefore be one of the mechanisms by which bran-fried AR enhanced the therapeutic effect.

In this study, we improved the understanding of the mechanisms through which AR treats SDS. Furthermore, we revealed possible mechanisms by which bran-fried AR enhances its therapeutic effect by comparing the modulatory effect on intestinal microbiota of raw and bran-fried AR in a rat model of SDS. However, the material basis of this modulatory effect remains unclear. In recent years, a large number of literatures have reported that polysaccharides in TCM can improve intestinal microbiota [39,40]. Liu et al. [41] determined the content of polysaccharides in ARs from different areas and found that most ARs were rich in polysaccharides. It is worth studying whether the abundant polysaccharides in AR and the specific changes of polysaccharide components in bran-fried AR are related to the regulation of intestinal microbiota. This will be the focus of our next research.

## 5. Conclusions

Our findings suggest that the biological mechanism by which AR treats SDS may be related to the modulation of intestinal microbiota. The mechanism by which bran-fried AR enhanced the therapeutic effect could be associated with improved modulation on *Bacteroides*, *Escherichia-Shigella*, *Phascolarctobacterium*, *Incertae-Sedis (Defluviitaleaceae Family)* and *Incertae-Sedis (Erysipelotrichaceae Family)*. These findings can help our understanding of the role and significance of bran-fried AR. In addition, the research method of this experiment can provide new ideas for the study of mechanism of action and processing principle of TCM.

## Figures and Tables

**Table 1:** The abundance of bacteria closely related to SDS among diverse groups.

Name	CG (10^−3^/%)	MG (10^−3^/%)	RA (10^−3^/%)	BFA (10^−3^/%)
Ruminococcus	436.025 ± 163.965	4010.280 ± 1610.945	625.017 ± 99.849 *	571.414 ± 139.984 *
Bacteroides	81.152 ± 19.609	1116.391 ± 275.562	168.912 ± 25.552 *	51.197 ± 11.961 *^Δ^
Parasutterella	45.710 ± 10.285	1.632 ± 0.528	45.882 ± 5.340 *	9.010 ± 2.544 *^Δ^
Escherichia-Shigella	1.397 ± 0.405	203.602 ± 42.451	21.553 ± 3.667 *	2.810 ± 1.212 *^Δ^
Phascolarctobacterium	0	1924.348 ± 228.220	32.973 ± 7.846 *	0.327 ± 0.126 *^Δ^
Enterobacter	0.604 ± 0.433	30.883 ± 14.957	0.293 ± 0.182 *	0 *
Acinetobacter	0	35.678 ± 17.171	0 *	0 *
Incertae_Sedis (Defluviitaleaceae Family)	13.682 ± 5.953	58.341 ± 24.318	98.844 ± 45.533 *	34.307 ± 15.597 *^Δ^
Incertae_Sedis (Erysipelotrichaceae Family)	1.005 ± 0.691	55.507 ± 23.189	72.524 ± 34.369 *	7.281 ± 5.162 *^Δ^

Values are presented as the mean ± standard deviation (*n* = 8). Differences were assessed by Kruskal–Wallis *H* test. ^*^ *p* < 0.05 when compared with MG; ^Δ^ *p* < 0.05 when compared with RA.

## Abbreviations

AR, Atractylodis Rhizoma; TCM, traditional Chinese medicine; SDS, spleen-deficiency syndrome; rDNA, ribosomal DNA; SPF, Specific pathogen Free; SD, Sprague Dawley; TNF-α, tumor necrosis factor-α; IL-6, interleukin 6; IgG, immunoglobulin G; MTL, motilin; GAS, gastrin; TRY, trypsin; AMS, amylase; SS, somatostatin; ELISA, Enzyme-linked immunosorbent assay; OTU, operational taxonomic unit; PCoA, Principal Co-ordinates Analysis; LDA, linear discriminant analysis; LEfSe, linear discriminant analysis effect size.
